# Hydrogen evolution reaction following the Slater–Pauling curve: acceleration of rate processes induced from dipole interaction between protons and ferromagnetic catalysts†

**DOI:** 10.1039/d2ra07865a

**Published:** 2023-04-25

**Authors:** Masao Morishita, Yuki Okumura, Ramu Fukushima, Hiroaki Yamamoto, Hidefumi Yanagita

**Affiliations:** a Department of Chemical Engineering and Materials Science, University of Hyogo 2167 Shosha Himeji 671-2201 Japan; b Sanalloy Industry Co., Ltd. 290-44 Takahashi Fukusaki-cho Kanzaki 679-2216 Japan morisita@eng.u-hyogo.ac.jp

## Abstract

Developing new concepts to design noble-metal-free catalysts is necessary to achieve the hydrogen economy and reduce global CO_2_ emissions. Here, we provide novel insights into the design of catalysts with internal magnetic fields by investigating the relationship between the hydrogen evolution reaction (HER) and the Slater–Pauling rule. This rule states that adding an element to a metal reduces the alloy's saturation magnetization by an amount proportional to the number of valence electrons outside the d shell of the added element. We observed that rapid hydrogen evolution occurred when the magnetic moment of the catalyst was high, as predicted by the Slater–Pauling rule. Numerical simulation of the dipole interaction revealed a critical distance, *r*_C_, at which the proton trajectory changes from a Brownian random walk to a close-approach orbit towards the ferromagnetic catalyst. The calculated *r*_C_ was proportional to the magnetic moment, consistent with the experimental data. Interestingly, *r*_C_ was proportional to the number of protons contributing to the HER and accurately reflected the migration length for the proton dissociation and hydration and the O–H bond length in water. The magnetic dipole interaction between the nuclear spin of the proton and the electronic spin of the magnetic catalyst is verified for the first time. The findings of this study will open a new direction in catalyst design aided by an internal magnetic field.

## Abstract 2

The magnetic dipole interaction between the nuclear spin of the proton and the electronic spin of the magnetic catalyst is verified for the first time. The findings of this study will open a new direction in catalyst design aided by a magnetic field.

## Graphical abstract

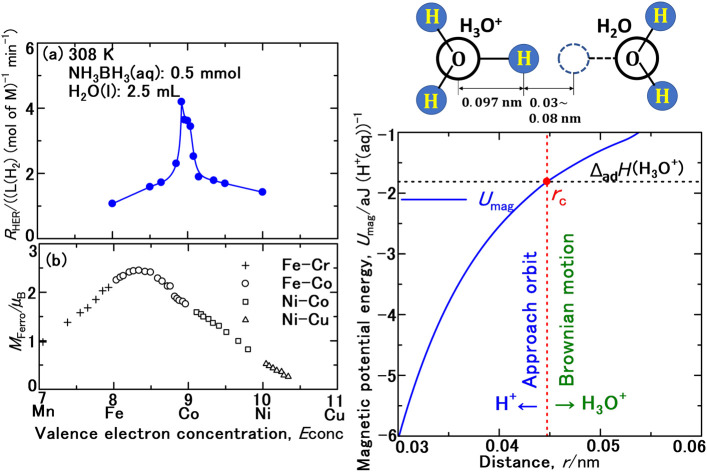

## Introduction

To reduce CO_2_ emissions, the production of hydrogen fuel instead of fossil fuels is critical.^1^ Although Pt-based catalysts are being widely used for hydrogen production,^2–8^ Pt is one of the most expensive noble metals. One strategy to achieve the hydrogen economy is to develop inexpensive catalysts with pertinent activity to replace Pt. In 1845, Faraday^9^ had an insight that magnetic forces affect electrolytes. However, his insight was greatly overlooked for the next 150 years. Recently, the use of magnetic fields has been highlighted as an effective strategy for improving the performance of catalysts for the oxygen evolution reaction (OER)^10–16^ and hydrogen evolution reaction (HER)^17–21^ during water splitting,^10–15,17–21^ and for enhancing power generation with fuel cells.^16^

The oxygen molecule, O_2_(g), has a triplet spin configuration. Catalysts with spin selectivity^22^ are known to improve OER efficiency by effectively inducing a triplet spin configuration, thereby decreasing the OER overpotential.^10–15^ The spin-selective reaction that accelerates OER consists of the following three steps:^10–16^ (I) spin-selective conduction in the catalyst acting as a spin filter;^11–15^ (II) spin transport from the catalyst to O_2_(g) to configure the triplet spin state;^12–16^ and (III) dissociation of triplet O_2_(g).^10^

Step (I) has been validated experimentally.^11–15^ TiO_2_ electrodes coated with self-assembled DNA,^11^ chiral paramagnetic CoO_*x*_ films,^14^ and ferrite oxides^13^ were shown to act as spin filters for OER. Concerning step (II), Gracia^12^ used transition theory to describe the tunnelling phenomenon as the mechanism of catalyst-to-O_2_(g) spin transport required to reach the spin-parallel triplet state. In addition, the spin-selective emission of β rays (*i.e.*, electron beams) was observed during the β decay of radionuclide ^60^Co.^23^ Regarding step (III), first-principles calculations verified that the triplet spin state is advantageous for the dissociation process.^10^

Exposure to an external magnetic field is found to increase the electric current generated during the HER.^17–21^ Water is composed of the diamagnetic H_2_O(l) molecule and charged ions, *i.e.*, proton (H^+^(aq)), hydroxide (OH^−^(aq)), and hydronium (H_3_O^+^(aq)) ions. Under an external magnetic field, as is well-known in the field of magnetohydrodynamics (MHD), an increase in the hydrogen evolution rate results from convection due to the Lorenz force^17–21^ and from enhanced electrical conductance due to the magneto-resistive effect.^20,21^ Nonetheless, the interaction between the nuclear spin of the proton and the electronic spin of the metal catalyst remains unclear.^9–22^

Tungsten carbide (WC) has 10 valence electrons, 5d^6^ from W and 2p^4^ from C, making it similar to Pt (5d^10^). In 1973, Levy and Boudart^24^ proposed that WC exhibited singular catalytic activity similar to Pt. Since then, many researchers have investigated this theory using spectroscopy,^25–27^ first principles calculations,^28,29^ and experiments to measure catalytic activity.^24,30–35^ The catalytic activities of WC and its composites have only been observed under applied external voltage,^30–35^ and the predicted intrinsic catalytic activity of mono-WC under voltage-free operation has not yet been verified.

Bennett *et al.*^25^ reported that magnetic properties are one of the intrinsic differences between WC and Pt. WC is non-magnetic, whereas Pt exhibits high magnetic susceptibility. In our previous study,^36^ an HER catalyst was developed by doping tungsten carbide (WC) lattice with ferromagnetic Co nanocrystals. The resulting alloy was used for the catalytic hydrolysis of ammonium borane (NH_3_BH_3_), a material known as a high-capacity hydrogen-storage compound.^37–43^ The activity of the novel carbide was 30% higher than Pt nanoparticles under the same conditions. We hypothesised that the enhanced catalytic activity was attributed to the synergistic effect of the WC matrix promoting hydrolytic cleavage of NH_3_BH_3_ and the ferromagnetic Co crystals interacting with the nucleus spin of the protons. In the present study, we aimed to verify the interaction between the nucleus proton and magnetic substances from both experimentation and numerical simulation. The relationship between the rate of HER and the magnetic moment of a catalyst was investigated considering the Slater–Pauling rule.^44,45^ This rule states that adding an element to a metal reduces the alloy's saturation magnetization by an amount proportional to the number of valence electrons outside the d shell of the added element. To understand the relationship between HER and the Slater–Pauling rule,^44,45^ the dipole interaction^44^ between the proton nucleus spin and magnetic substances was simulated based on the electromagnetism for the first time.

An application of HER catalysts is the generation of hydrogen fuel from NH_3_BH_3._ In its stable crystal form, NH_3_BH_3_ contains 19.6 wt% hydrogen,^37–43^ and is being investigated for efficient transportation of hydrogen-based fuel for portable fuel-cell systems. Previous studies investigated the HER by hydrolysis over 10 wt% Co (ref. 39) or 2 wt% Pt (ref. 40) (both supported by Al_2_O_3_) and found that the HER in the latter was significantly faster than that in the former. A similar HER in NH_3_BH_3_(aq) catalysed by Ni nanoparticles (NPs) supported by a zeolite molecular sieve was observed.^42^ The atomic configuration in the AB molecule was investigated by neutron diffraction.^38^ The chemical bonding states for AB were investigated by first principles calculations^41^ and soft X-ray adsorption spectroscopy.^43^ The standard enthalpy of formation, 
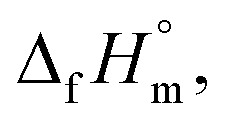
 at 298.15 K was determined by combustion calorimetry.^37^ The catalytic design of selecting metals and alloys based on thermodynamic cycles for hydrolysis of AB have not previously been investigated. In the present study, a new strategy was developed to enhance catalytic performance and investigate the effect of an internal magnetic field on the thermodynamic mechanism of the hydrolysis of AB.

## Methods

### Sample preparation

Cobalt(ii) acetate tetrahydrate [Co(C_2_H_3_O_2_)_2_·4H_2_O, 99%, Kojundo Chemical Laboratory Co., Ltd., Saitama], iron(iii) nitrate nonahydrate [Fe(NO_3_)_2_·9H_2_O, 99.9%, Kojundo Chemical Laboratory Co., Ltd., Saitama], and nickel(ii) acetate tetrahydrate [Ni(C_2_H_3_O_2_)_2_·4H_2_O, 99%, Kanto Chemical Co., Inc., Tokyo], were used as the starting materials. Thirteen catalyst compositions were prepared with various compositions using the following procedure. For each sample, the requisite amount of Co(C_2_H_3_O_2_)_2_·4H_2_O, Fe(NO_3_)_2_·9H_2_O, and Ni(C_2_H_3_O_2_)_2_·4H_2_O were separately dissolved in 100 ml deionized water at 363 K contained in alumina crucibles.

For all samples (except 1, 7, and 13), the corresponding precursor solutions were mixed to form homogeneous aqueous solutions with a total volume of 200 ml. The homogeneous mixtures of metal complexes were prepared by calcination at about 500 K. These mixtures were then thermally decomposed under O_2_ at 773 K for 2 h to obtain homogeneous oxide-containing components. After thermal decomposition, powder samples were produced by reducing oxide-containing components with H_2_ at 1073 K for 2 h followed by cooling with 20 K min^−1^. Using an electron probe microanalysis system (JXA-8530FPlus, Co., JOEL Ltd, Tokyo) with a 15 kV accelerating voltage, the homogeneous chemical compositions of the samples were confirmed *via* X-ray images. Particle sizes and morphologies were compared by scanning electron microscopy (SEM).

The *SA* of the samples was determined by the Brunauer–Emmett–Teller method using nitrogen physisorption isotherms at 77 K, obtained with a sorption and porosity analyzer (BELSORP mini, MicrotracBEL Corp). The SA of the sample powders was about 2 m^2^ g^−1^, as shown in Table 1.

**Table 1:** Composition, electron concentration (*E*_conc_), magnetic moment (*M*_Ferro_),^44,45^ specific surface area (SSA), structure determined from XRD, average electron-vacancy number, *N*_v_,^54^ and the *d* level of alloying element, *M*_d_^55^

Sample	Co/mol%	Fe/mol%	Ni/mol%	*E*_conc_ a	*M*_Ferro_/*μ*_B_^b^	SSA/(m^2^ g^−1^)	Structure	*N*_v_	*M*_d_/eV
1	0	100	0	8.00	2.20	2.01	bcc	2.66	0.81
2	50	50	0	8.50	2.29	1.92	bcc	2.19	0.43
3	65	35	0	8.65	2.23	2.27	bcc	2.04	0.32
4	85	15	0	8.85	1.87	1.71	fcc^c^	1.85	0.17
5	92	8	0	8.92	1.82	1.94	fcc	1.79	0.12
6	96	4	0	8.96	1.76	1.94	fcc^d^	1.75	0.09
7	100	0	0	9.00	1.70	1.93	hcp	1.71	0.06
8	96	0	4	9.04	1.66	1.92	hcp + fcc^e^	1.67	0.09
9	92	0	8	9.08	1.59	1.86	hcp + fcc^e^	1.63	0.13
10	85	0	15	9.15	1.55	1.91	hcp + fcc^e^	1.55	0.18
11	65	0	35	9.35	1.37	1.85	fcc	1.28	0.27
12	50	0	50	9.50	1.18	1.47	fcc	1.19	0.48
13	0	0	100	10.0	0.60	1.93	fcc	0.66	0.89

a The *E*_conc_ values for Fe (3d^6^ and 4s^2^), Co (3d^7^ and 4s^2^), and Ni (3d^8^ and 4s^2^) are 8, 9, and 10, respectively. The Slater–Pauling rule^44,45^ was used to calculate the *E*_conc_ of alloys by averaging the *E*_conc_ of the elemental components.

b The *M*_Ferro_ values for the samples (except 1, 7, and 13) were estimated by interpolating the Slater–Pauling curve.

c Two phase equilibrium region composed of fcc and bcc solid solutions in the Co–Fe binary system.^46^

d Single phase region composed of hcp solid solutions in the Co–Fe binary system.^46^

e Single phase region composed of hcp solid solutions in the Co–Ni binary system.^46^

## HER analysis

For HER tests, 20 mg of each sample was placed in a glass test tube containing 1 ml of H_2_O. Separately, solutions of NH_3_BH_3_(aq) were made by dissolving 0.5 mmol NH_3_BH_3_(cr) in 1.5 ml H_2_O. This solution was then mixed with each sample to initiate hydrolysis and hydrogen evolution. The volume of evolved hydrogen (*V*_HER_) was measured as a function of time *t*, and the hydrogen evolution rate (*R*_HER_) was determined from the slope of the *V*_HER_*vs. t* curve. From this, it was determined that *V*_HER_ increased linearly while excess unreacted NH_3_BH_3_(aq) remained. The used aqueous solution was decanted after the first *V*_HER_ measurement, while the ferromagnetic samples were held back inside the test tube with an external magnet placed outside the glass test tube. After draining, the second and third *V*_HER_ measurements were conducted similarly to the first. After completing the third measurement, fresh water was added to the ferromagnetic samples, which were then drained. After drying the samples in a dryer overnight, a fourth measurement was performed in the same way. The fourth *V*_HER_ measurement was similar to the first three. Because the produced hydrogen gas appeared to reduce the sample surface, the *V*_HER_ obtained in all four was averaged and used as the total *V*_HER_. During the HER test, the AB solution was stirred with a rotating magnetic stirrer.

## Results and discussion

### Hydrogen evolution following the Slater–Pauling magnetic rule

The structures of the samples determined from XRD are summarized in Table 1. The phases were consistent with the phase diagrams^46^ of the Co–Fe and Co–Ni binary systems. However, the phases composed in 85 mol% Co–15 mol% Fe (no. 4), 96 mol% Co–4 mol% Fe (no. 6), 96 mol% Co–4 mol% Ni (no. 8), 96 mol% Co–8 mol% Ni (no. 9), 85 mol% Co–15 mol% Ni (no. 10) were different from the equilibrium phase diagrams^46^ as shown in Fig. S1–S5 (ESI†). In no. 4, the high temperature fcc solid solution^47^ was only formed due to rapid cooling (20 K min^−1^), which prevented the formation of the equilibrium bcc solid solution.^46^ In no. 6, the high temperature fcc solid solution^47^ remained due to rapid cooling, which prevented the formation of the equilibrium hcp solid solution.^48^ In no. 8–10, the high fcc solid solution^47^ remained partly mixed with the equilibrium hcp solid solution^48^ resulting from rapid cooling. However, the meta stable fcc solid solutions were in the ferromagnetic phase.

**Fig. 1:**
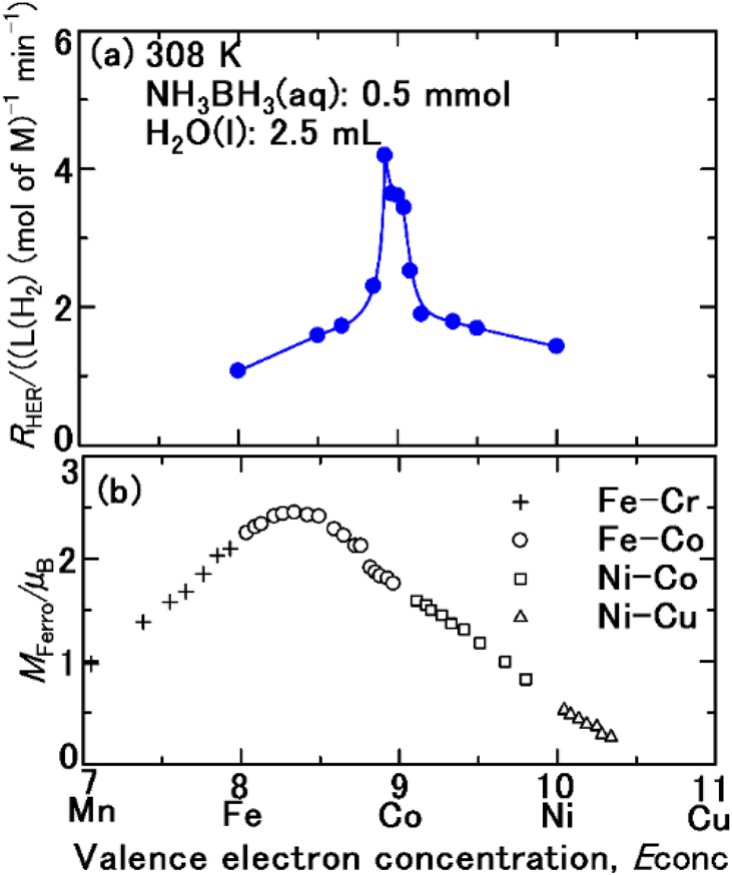
(a) Hydrogen evolution rate *R*_HER_ for NH_3_BH_3_(aq) over pristine Fe, Co, and Ni, and their alloys as a function of the valence electron concentration, *E*_conc_. (b) Slater–Pauling curve showing the relationship between the magnetic moment *M*_Ferro_ of pristine metals or their alloys and *E*_conc_.

Fig. 1a and b compare the plots of *R*_HER_ for the hydrolysis of ammonium borane (NH_3_BH_3_)^36^ as a function of the valence electron concentration *E*_conc_, of the catalyst and the Slater–Pauling curve.^44,45^ The composition, *E*_conc_, magnetic moment, SSA, and crystal structure of Fe, Co, Ni, and their binary alloys are shown in Table 1. The perpendicular axis of the Slater–Pauling curve^44,45^ is the magnetic moment *M*_Ferro_ with the units of Bohr magneton. The *M*_Ferro_ of pure Fe, Co, and Ni, as well as their alloys, varies with *E*_conc_. The Co–Fe alloy, with an *E*_conc_ of 8.33, has the highest *M*_Ferro_. The *R*_HER_ increases with *E*_conc_, beginning with Fe (*E*_conc_ = 8), and reaching a maximum for the 92Co–8Fe (mol%) alloy with *E*_conc_ = 8.92. As *E*_conc_ continues to increase, *R*_HER_ decreases. Specifically, *R*_HER_ varies according to the Slater–Pauling curve, and a magnetic-moment-induced increase in *R*_HER_ is observed.

To clarify the reason for that the magnetic-moment increase in *R*_HER_, the first of all, the thermodynamic cycle of hydrolysis of AB was discussed. Table 2 shows the thermodynamic cycle of the hydrolysis of AB where the standard enthalpies of formation, 
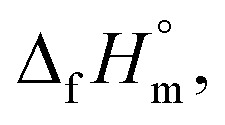
 at 298.15 K of the standard substances of NH_3_BH_3_(cr),^37^ H_2_O(l),^49^ orthoboric acid (B(OH)_3_(aq)),^49^ ammonium (NH^4+^(aq)),^49^ metaboric acid (BO_2_^−^(aq)),^50^ and H_2_(g)^49^ are summarised in Table S1.† Eqn (I) shows the hydration reaction of NH_3_BH_3_(cr), where the thermodynamic value is unknown. Eqn (II) shows the HER of the hydrolysis of NH_3_BH_3_(aq). Eqn (III), rewritten as the sum of eqn (I) and (II), indicates the HER from the initial substance of NH_3_BH_3_(cr). Eqn (IV) shows the formation of NH^4+^(aq). Eqn (V) shows the formation of BO_2_^−^(aq). Finally, eqn (VI), rewritten as the sum of eqn (III)–(V), shows the final state of the hydrolysis Since eqn (IV)–(VI) are spontaneous reactions, the HER is given by eqn (III). As the standard entropy, 
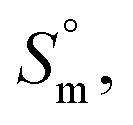
 of NH_3_BH_3_(cr) has not yet been measured, the standard entropy of reaction, Δ_r_*S*^°^, and the standard Gibbs energy of reaction, Δ_r_*G*^°^, are unknown. However, Δ_r_*G*^°^ is more negative than Δ_r_*H*^°^ as the HER increases Δ_r_*S*^°^. Therefore, when a driving energy is applied corresponding to the hydrogen overpotential of metals, the HER reaches equilibrium, as defined by eqn (VI) *via* eqn (III).

**Table 2:** Thermodynamic cycle of hydrolysis of ammonium borane, NH_3_BH_3_

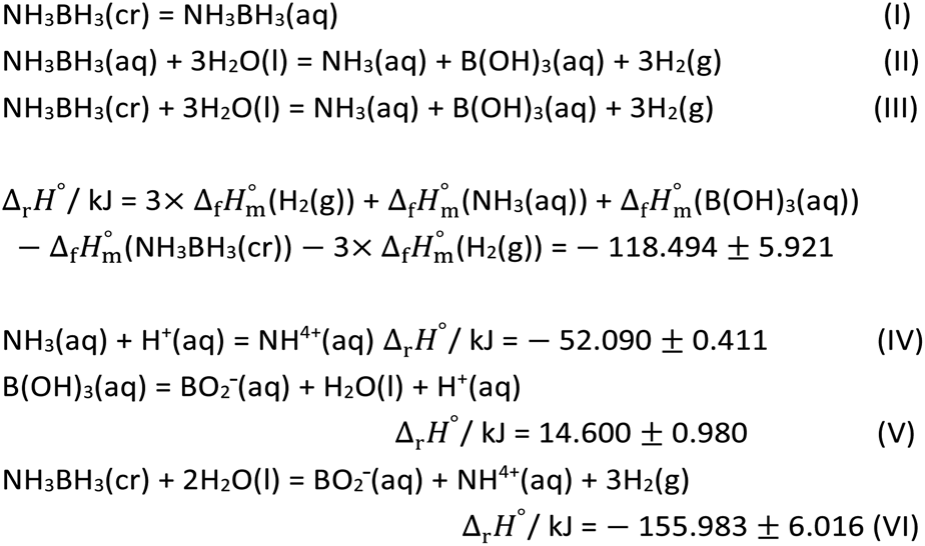

**Fig. 2:**
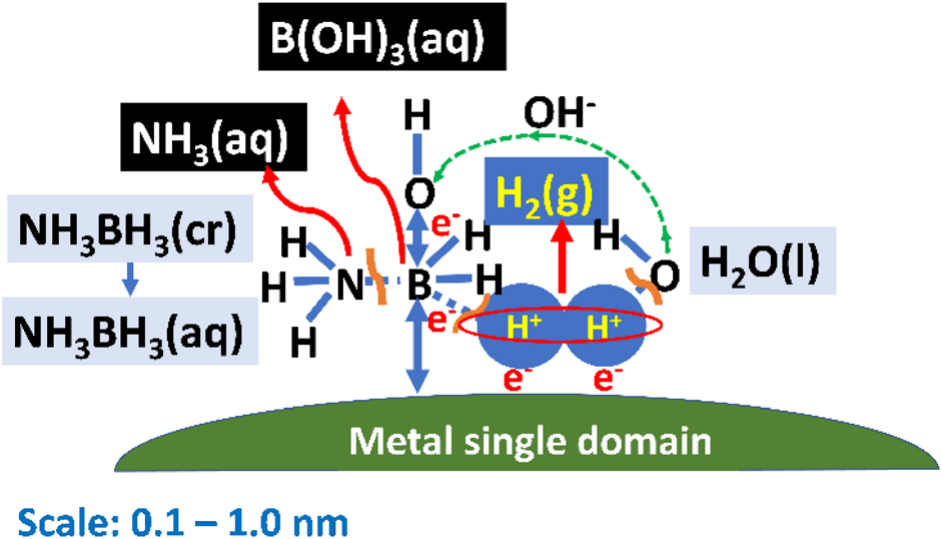
Schematic of hydrogen evolution *via* the hydrolysis of NH_3_BH_3_(aq) over a magnetic metal single domain. A sufficient number of protons from BH_3_(aq) and H_2_O(l) assemble at the single domain owing to the magnetic interaction between their nuclear spins and the electronic spin of the single domain.

Next, we sought to understand the HER mechanism using the Slater–Pauling rule.^44,45^Fig. 2 depicts a schematic illustration of the catalytic HER over a magnetic metal single domain, which is relevant for Fe, Co, and Ni pure metals and their alloys. The HER is carried out through the following steps: (1) NH_3_BH_3_ molecules collide during Brownian motion^51^ and are adsorbed on the single domain. (2) The B–N bonds in NH_3_BH_3_(aq) are broken to form the stable NH_3_(aq) molecule and transition-state species BH_3_(aq) to reach equilibrium. (3) Short-lived BH_3_(aq) releases three quasi-stable protons, and the B atom coordinates the three OH^−^(aq) ions released from the surrounding H_2_O(l) molecule to form a stable B(OH)_3_ molecule. H_2_O(l) also releases three protons. (4) A sufficient number of protons from BH_3_(aq) and H_2_O(l) assemble at the single domain owing to the magnetic interaction between their nuclear spins and the electronic spin of the single domain. (5) Hydrogen molecules are produced when protons accept electrons (e^−^). (6) One e^−^ is released, along with a proton, during the decomposition of BH_3_, while the other e^−^ is released from OH^−^(aq) during coordination to form B(OH)_3_(aq). In other words, the source of charge transfer is the B atom adsorbed on the metal single domain. It is likely that an attractive dipole interaction^44^ occurs directly between a proton and ferromagnetic single domain when the nuclear and electronic spins of the catalyst are aligned in parallel. As a result, the magnetic force of a ferromagnetic material interacts with protons followed by H_2_ (g) evolution. An attractive dipole interaction^44^ was studied by a numerical simulation in the next section.

The thermodynamic cycle shown in Table 2 can be re-written as an electrochemical cycle, given by

1 Anode: NH_3_NH_3_(aq) + 3H_2_O(l) = NH_3_(aq) + B(OH)_3_(aq) + 6H^+^(aq) + 6e^−^

2 Cathode: 6H^+^(aq) + 6e^−^ = 3H_2_(g)

Therefore, the cathodic protection mechanism prevents the surface of the sample from corrosion, which is further evidence for HER following the Slater–Pauling magnetic rule.^44^

**Fig. 3:**
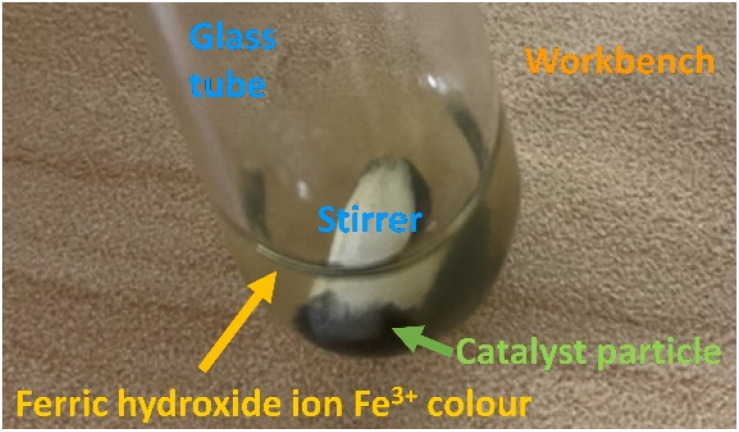
Appearance of 85 mol% Co–15 mol% Fe sample after hydrolysis of ammonia borane. The aqueous solution shows orange colour indicating that Fe^3+^ aqueous ion was formed.

The maximum *M*_Ferro_ in the Slater–Pauling curve is at *E*_conc_ = 8.33, whereas the maximum *R*_HER_ is at *E*_conc_ = 8.92. That is, the HER drop was observed in the samples including 15 mol% Fe (no. 4), 35 mol% Fe (no. 3), 50 mol% Fe (no. 2), and pristine Fe (no. 1). The cause of the HER drop was examined. The NH_3_BH_3_(aq) solution was alkaline (pH 8.53). The electric potential (*E*_h_)–pH diagram^52^ indicates that Fe_2_O_3_(cr) is stable. Therefore, the aqueous ion equilibrated with Fe_2_O_3_(cr) is ferric hydroxide ion Fe^3+^(aq), which appears with an orange colour. Fig. 3 shows the aqueous solution after first series of the hydrolysis of AB over the sample 85 mol%–15 mol% Fe (no. 4). Orange colour development was clearly observed. The same colour development occurred with other samples including 35 mol% Fe (no. 3), 50 mol% Fe (no. 2), and pristine Fe (no. 1). Consequently, the corrosion of samples with over 15 mol% Fe resulted in overriding of the cathodic protection (eqn (2)).

It is well known that d-electron vacancies^53^ have control on the corrosion of transition metals and alloys. d-electron vacancies^53^ capture the electrons of OH^−^(aq), resulting in adsorption of the radical oxygen atoms. The alloys with high Fe content have many electron vacancies in the 3d band. The samples with over 15 mol% Fe (no. 1–4) had many d-electron vacancies, which adsorb the radical oxygen atoms to form a carrion product, Fe_2_O_3_. This product overrides the cathodic protection (eqn (2)) during the hydrolysis of AB, which was concluded to be the reason for the HER drop.

The quantitative contents of the d-electron vacancies^53^ were defined as the *N*_v_ values.^54^ The phase stabilities of the super heat resistant alloys are estimated in terms of the *N*_v_ values. The alloys with excess *N*_v_ values form harmful *σ* phases, as was determined by multiple regression analysis^54^ of the experimental data. The optimum alloy compositions are simulated to be less than the critical *N*_v_ value, known as PHACOMP (Phase Computation).^54^ The d-orbital level parameter, *M*_d_, was suggested based on the molecular orbital calculations to update the *N*_v_ values considering the alloying effect.^55^ The *N*_v_ (ref. 54) and *M*_d_ (ref. 55) values for the samples are shown in Table 1.^54,55^ The critical value of *N*_v_ was 1.85 and *M*_d_ was 0.17 eV for 85 mol% Co–8 mol% Fe (no. 4), which was determined to prevent corrosion. When values less than the critical values are used, HER is actively caused by the cathodic protection of the surface from corrosion.

### Numerical simulation of the dipole interaction between proton and magnetic catalyst

A hypothesis that an attractive dipole interaction occurs directly between a proton and ferromagnetic single domain when its nuclear and the electronic spin of the catalyst are aligned in parallel based on that HER follows the Slater–Pauling rule. In this section, the dipole interaction^44^ was directly investigated by numerical simulation.

**Fig. 4:**
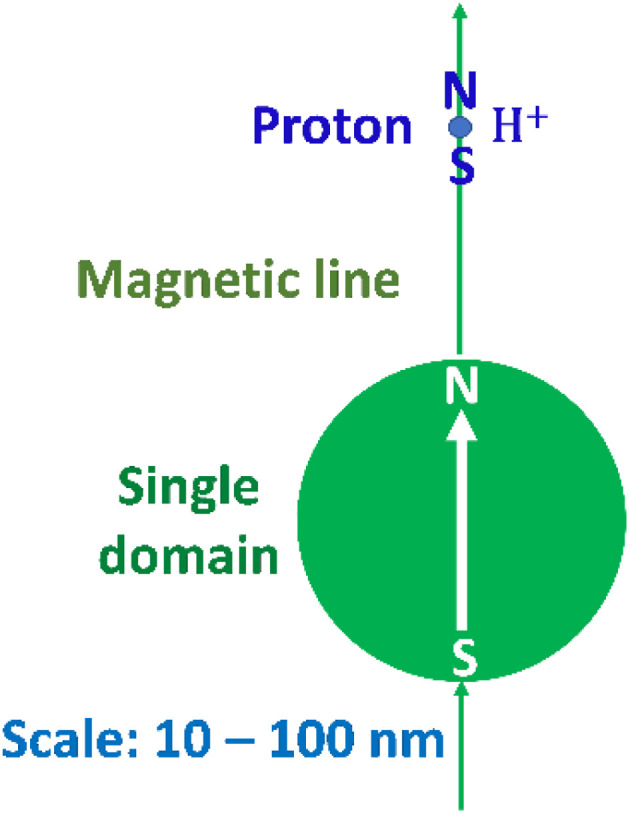
Schematic of the dipole interaction between a proton and ferromagnetic nano sphere single domain.^56–59^

Fig. 4 depicts a schematic of the most fundamental model.^44^ The potential energy resulting from this magnetic dipole interaction *U*_mag_, is defined as follows.3
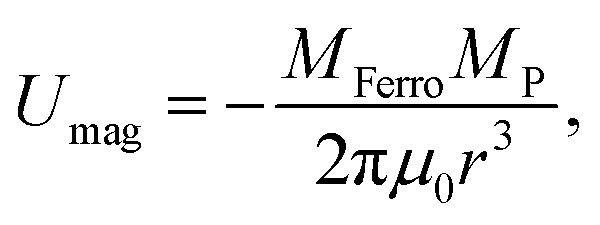
where *M*_Ferro_ and *M*_P_ are the magnetic moments of the nano sphere single domain,^56–59^ and the proton, respectively, *μ*_0_ is the permeability of free space, 4π × 10^−7^ (H m^−1^).^44^ The *r* datum is the distance between the N and S, or S and N poles of them. Accordingly, the magnetic force, *F*, exerted by the ferromagnetic single domain to the proton is defined as follows.4
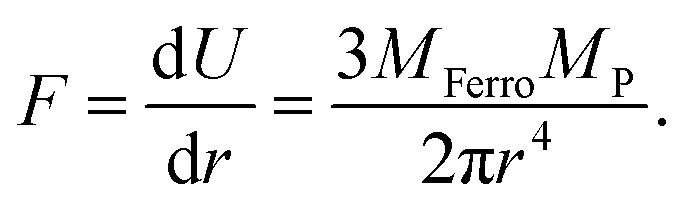


The acceleration, *a*, of the proton is determined by dividing *F* by the mass of the proton *m*_P_ (1.67262171 × 10^−27^ kg).^60^5
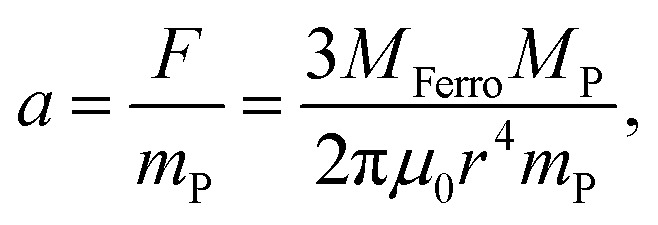


The present model includes a Co single domain sphere with an assumed diameter, *d*, of 60 nm to correlate it with the same domain in the WC lattice used in our previous study.^36^ The magnetic moment per Co atom^44,45^ is 1.7*μ*_B*,*_ and the number of moles, *n*, in this Co single domain is 1.6945 × 10^−17^ based on the density of hcp Co (8.9 Mg m^−3^).^61^ Therefore, the number of atoms, *N*, is 1.0205 × 10^7^. The magnetic moment of the Co single domain is *N* × 1.7*μ*_B_ per Co atom;^44,45^ hence, *M*_Ferro_ equals 2.0211 × 10^−22^ Wb m. The *M*_P_ of a proton^44^ is 6.33 × 10^−33^ Wb m.

**Fig. 5:**
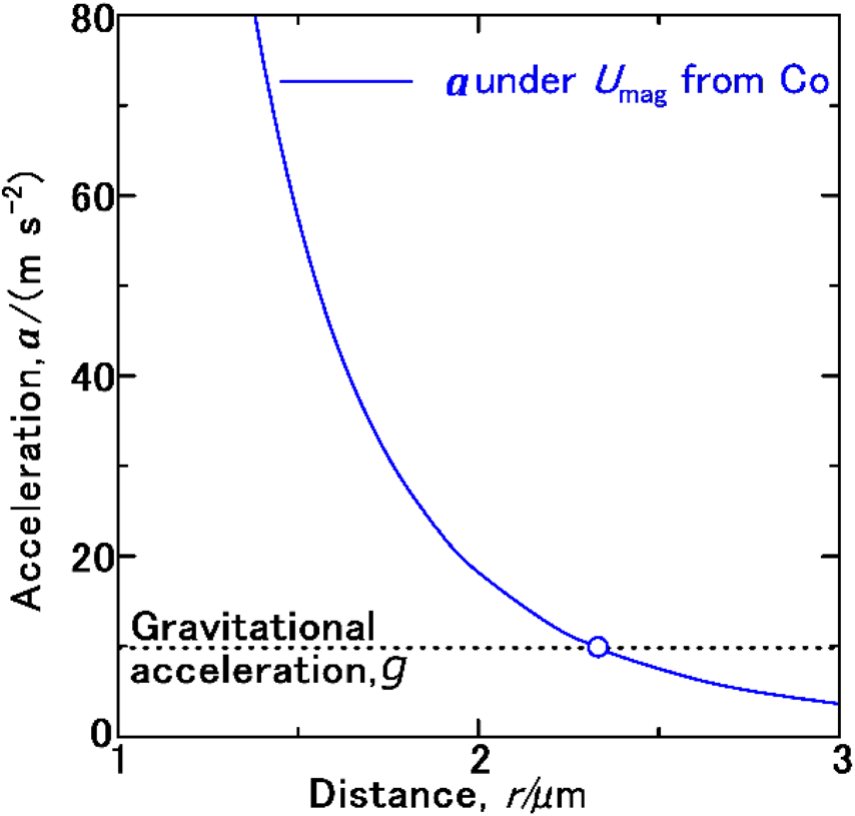
Acceleration, *a*, acting on a proton as a function of the distance *r* from a Co single domain with a diameter of 60 nm under the magnetic potential energy *U*_mag_ acting on a proton.

These *M*_Ferro_ and *M*_P_ values were used in eqn (3) to simulate *a* as a function of *r*, and the resulting plot is shown in Fig. 5. When *r* is 2.33375 μm, *a* is 9.807 ms^−2^, consistent with the gravitational acceleration, *g*.^60^ Because *g* was obtained at the appropriate distance, the present simulation accurately estimated the magnetic dipole interaction.

**Fig. 6:**
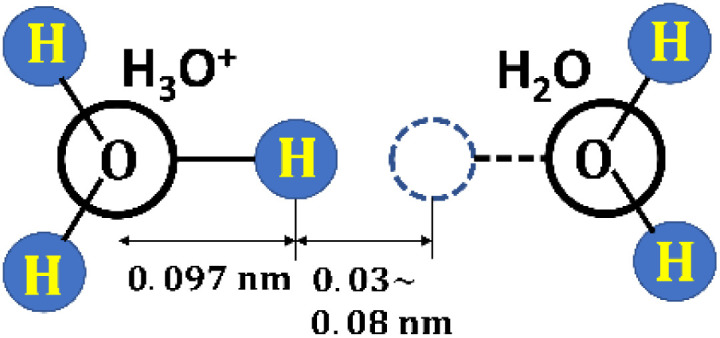
Schematic of the structures of hydronium ion, H_3_O^+^(aq), and H_2_O(l) molecules, and migration length of proton between them.^62–64^

To confirm that the proton is attracted to the magnetic force of the ferromagnetic single domain, its hydration was subsequently investigated. The hydration enthalpy Δ*H*^+^_ad_ is −260.7 ± 2.5 (kcal mol^−1^),^62,63^*i.e.*, −1.811 [aJ (H^+^_ad_)^−1^]^62,63^ per proton. With the Brownian motion,^29^ a proton dissociates from H_3_O^+^(aq) and is then hydrated by another H_2_O(l) molecule.^64–66^Fig. 6 shows schematic structures for H_3_O^+^(aq) and H_2_O(l) molecules, and a proton migrating between them. The migration length *l*_m_ of the dissociation and hydration is 0.03–0.08 nm.^64^ Moreover, the O–H bond length in the H_2_O(l) molecule is 0.097 nm.^64^

**Fig. 7:**
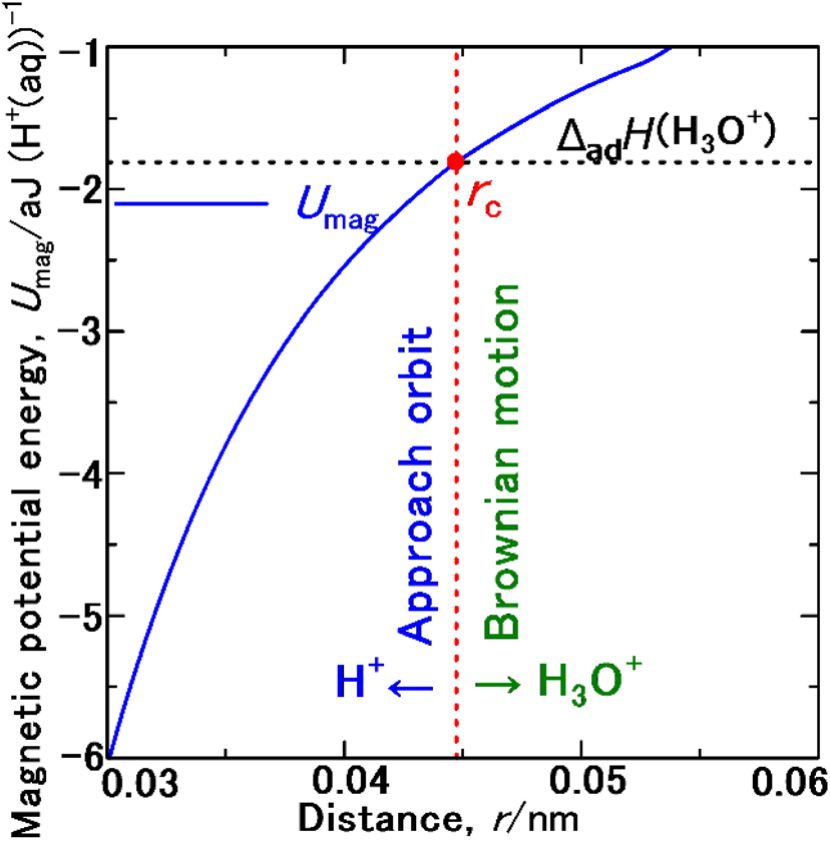
*U*_mag_ acting on a proton as a function of *r* from a Co single domain with a diameter of 60 nm. *r*_c_ is the critical distance at which the proton, hydrated by a H_2_O molecule as H_3_O^+^(aq), changes its path from a Brownian random walking trajectory to a close-approach orbit towards the ferromagnetic single domain.

Fig. 7 shows the effect of *U*_mag_ of a Co single domain on a proton as a function of *r* (in the range of 0.03–0.06 nm) as described by eqn (3). With decreasing *r*, *U*_mag_ shifts to negative values. Remarkably, at 0.044725 nm, *U*_mag_ becomes equal to −1.811 [aJ (H^+^_ad_)^−1^],^62,63^ which is the Δ*H*^+^_ad_. The *r* value at which *U*_mag_ is equal to Δ*H*^+^_ad_ is the critical distance *r*_c_, exactly. Therefore, as shown in Fig. 7, when *r* < *r*_c_ (0.044725 nm), *U*_mag_ becomes deeper than Δ*H*^+^_ad_, causing a proton to be attracted to the Co single domain. Specifically, protons assemble on the Co single domain, and hydrogen gas rapidly evolves. (A) Initially, a proton accepts an electron to form a hydrogen atom, followed by adsorption (Volmer mechanism^2,3^). (B) Subsequently, another proton accepts another electron and aggregates with a hydrogen atom to form a dimer molecule, and a hydrogen gas molecule H_2_(g) desorbs (Heyrovsky mechanism^2,4^). (C) Selectively, two hydrogen atoms aggregate to form a dimer molecule, and a hydrogen gas molecule H_2_(g) dissociates (Tafel mechanism^5,6^).

In contrast, when *r* is greater than *r*_c_, *U*_mag_ becomes shallower than Δ*H*^+^_ad_, causing a proton to move towards an H_2_O molecule and form H_3_O^+^(aq) in Brownian motion. In conclusion, *r*_c_ is the exact distance at which the trajectory of a proton changes from a random walk caused by Brownian motion to an approach orbit towards the Co single domain. Moreover, the calculated *r*_c_ value (0.044725 nm) is within the range of *l*_m_ of proton dehydration and rehydration (0.03–0.08 nm^64,66^) and the O–H bond length (0.097 nm (ref. 62) in water. The effective particle number, *N*_eff_, of the proton, of which diameter, *l*_P_, is 8.751 × 10^−7^ nm,^67^ contributing to the frequency factor, *A*, in the Arrhenius equation^68^ is likely to be proportional to *r*_c_.

**Fig. 8:**
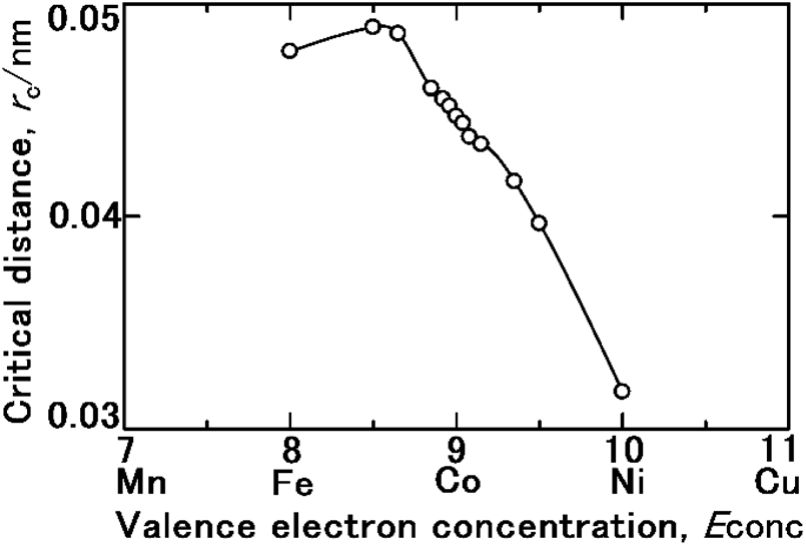
Critical distance *r*_c_ as a function of the valence electron concentration *E*_conc_. *r*_c_ is the distance at which a proton changes its path from a Brownian random walk trajectory to a close-approach orbit towards a single domain while hydrated by an H_2_O molecule as H_3_O^+^(aq). The diameter of each single domain is assumed to be 60 nm.

The *r*_c_ values for other single domains with the same compositions as the samples (Table 1) were calculated in the same way. Additionally, the *M*_Ferro_ values were estimated by interpolating the Slater–Pauling curve. Fig. 8 depicts the calculated *r*_c_ as a function of *E*_conc_, which shows that *E*_conc_ is optimal when *r*_c_ is 8.5. Theoretically, the contribution of *N*_eff_ to *R*_HER_ is the highest at *E*_conc_ = 8.5. However, as shown in Fig. 1, the maximum *R*_HER_ is obtained when *E*_conc_ is 8.92. Due to excess Fe, the formation of Fe_2_O_3_, resulting from excess 3d electron vacancies as above described, inhibited by the Heyrovsky^2,4^ and Tafel mechanisms,^5,6^ which are mediated by the Volmer mechanism.^2,3^

The auxiliary data for the present numerical simulation were summarized in Table 3.

**Table 3:** Auxiliary data of the permeability of free space (*μ*_0_), mass (*m*_p_) and diameter (*l*_P_) of the proton, magnetic moment of a Co atom (*β*), densities (*ρ*) of Co and WC, magnetic moment, (*M*_P_) of a proton, hydration enthalpy (Δ*H*^+^_ad_), gravitational acceleration (*g*), the adopted data of numbers of moles (*n*), and Co atoms (*N*), magnetic moment (*M*_Ferro_), in the 60 nm sphere Co single domain for the present study

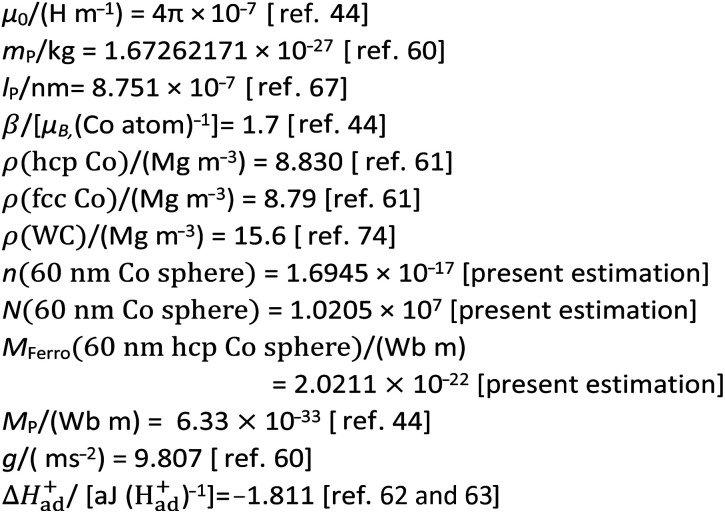

The singular catalytic activity of the Co nanocrystal doped WC in our previous study^36^ was discussed. The valence electron number of WC and Pt are the same. However, their magnetic properties are different and Pt has a higher magnetic susceptibility because WC is non-magnetic. Singular WC does not show catalytic activity. In our previous study,^36^ WC was doped with 60 nm diameter ferro-magnetic Co crystals to introduce an ordered-spin configuration, which showed a *R*_HER_ value even higher than that of the Pt nanoparticles during the hydrolysis of AB. A hypothesis for the enhanced catalytic activity was attributed to the synergistic effect of the WC matrix promoting hydrolytic cleavage of NH_3_BH_3_ and the ferromagnetic Co crystals interacting with the nucleus spin of the protons. The present verified attractive dipole interaction between protons and Co is evidence for the hypothesis of singular catalytic activity of WC arising from an internal magnetic field.

It was previously hypothesised that a synergetic effect of WC breaking NH_3_BH_3_(aq) to form protons and the antiparallel alignment of the nuclear spins of protons and electronic spins in a single domain to increase the magnetic entropy.^36,69,70^ Therefore, the 1s electronic spin of the hydrogen atoms absorbed by Pd (ref. 71) and adsorbed on Gd (ref. 72) induce disorder of their electron spin polarisation, thereby increasing the entropy of the system. By the above-described mechanism for HER, it is concluded the dipole attractive interaction between proton and magnetic catalyst enhances HER. After donating electrons by the Volmer mechanism,^2,3^ electronic spins of that hydrogen atoms are likely to be aligned against spins of Co. This problem should be further investigated by molecular orbital calculations.

AB is hopeful hydrogen fuel.^37–43^ The HER by hydrolysis over the 10 wt% Co nano particle supported on the SiO_2_ nano particle (Co/SiO_2_) was investigated by Xu and Chandra.^39^ In order to compare HER property of the Co particle obtained in the present study with one of Co/SiO_2_ by Xu and Chandra,^39^ their normalised hydrogen evolution rate per unit area (m^−2^), *R*^N^_HER_, was evaluated. Table 4 shows their *R*^N^_HER_ data. The experimental conditions for Co/SiO_2_ were 50 percent more conc. in the concentration of AB, 25 times larger in SSA (=52 m^−2^ g^−1^), and 83 percent smaller in mass (3.4 mg) than ones for the present Co particle. Considering difference in the experimental conditions, the intrinsic *R*^N^_HER_ data appears to be similar.

**Table 4:** Normalised hydrogen evolution rate per unit area, *R*^N^_HER_, of the Co particle in the present study, compared with the Co nano-crystal in WC matrix,^36^ and the 10 wt% Co nano particle supported on γ-Al_2_O_3_ nano particle.^39^

	*R* ^N^_HER_/(H_2_ mmol min^−1^ m^−2^)	*n*(AB)/mmol	*V*(H_2_O)/mL	Remarks
Co particle	1.28	0.5	2.5	This study
Co(cr)/SiO_2_	0.70^a^	3.2	10	Ref. 39
Co(cr) in WC	31.9^b^	0.5	2.5	Ref. 36

a SSA of the Co nano was 52 m^−2^ g^−1^, and its mass was 3.4 mg.

b SSA was hypothetically estimated as volume ratio of the Co nano-crystal against WC matrix.

The atomic configuration in the AB molecule was investigated by the neutron diffraction by Klooster *et al.*^38^ The chemical bonding states for AB were investigated by the first principles calculation by Banu *et al.*,^41^ and soft X-ray adsorption spectroscopy by Niibe *et al.*^43^ The 
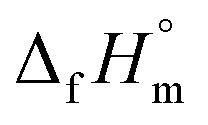
 datum at 298.15 K was determined by the combustion calorimetry by Shaulov.^37^ The effect of an internal magnetic field cooperating the cathodic protection on the thermodynamic mechanism of the hydrolysis of AB has been suggested in the present study for the first time.

In our previous study for the Co doped WC,^36^ the activation energy determined by the Arrhenius plots was found to be well consistent with the electrochemical hydrogen over potential of Co.^73^ This means hydrogen gas H_2_(g) is evolved over the Co crystals *via* Heyrovsky^2,4^ and Tafel mechanisms.^5,6^ The *R*^N^_HER_ datum of the Co crystals in the WC matrix was compared with that of the Co particle in the present study. Where the ratio of SSA of the fcc Co^36^ nanocrystals against the WC matrix were hypothetically assumed to be as same as the ratio of their volumes.^61,74^ Their volumes were calculated from their densities (Table 3). Table 4 shows their *R*^N^_HER_ data. The *R*^N^_HER_ of the Co crystal was found to be 25 times higher than that of the Co particle in the present study. This means that WC, of which electrons density of states are similar to Pt, adsorbs much amount of AB followed by decomposing the B–N bonding, and makes much protons dissociating from AB. Much protons are aligned on the Co crystals by the attractive dipole interaction, and 25 times higher *R*^N^_HER_ is concluded to be caused.

The parameters used in the present simulation are an extremely small scale power functions as, *e.g.*, *M*_Ferro_/(Wb m) = 2.0211 × 10^−22^, and *M*_P_/(Wb m) = 6.33 × 10^−33^ (Table 3) ^44^ the obtained *r*_c_ datum reflects the structure parameters for H_3_O^+^(aq) and H_2_O(l) molecules, and a proton migration (Fig. 6), indicating that the present numerical simulation was done with highly accuracy. Although a very fundamental model based on the sphere single domain magnetic structure^64–66^ was adopted, this is a novel finding and likely to give impact to general science due to that proton in aqueous solution are associated with various phenomena in nature. In the future, the dipole interactions in the complicated magnetic force lines from the magnetic substances composed of the multi domain should be investigated.

## Conclusions

The current study clarified the magnetic dipole interactions between the nuclear spin of a proton and the electronic spin of a magnetic catalyst for the first time. The conclusions are as follows: (1) the HER rate varied in accordance with the Slater–Pauling rule, resulting in a rapid rate of hydrogen evolution for catalysts with a high magnetic moment. (2) Numerical simulation of the magnetic dipole interaction revealed a critical distance at which the proton, as H_3_O^+^(aq), changes its path from a Brownian random walk trajectory to a close-approach orbit towards the ferromagnetic catalyst, in the same order as the migration length of the dissociation and hydration of the proton and O–H bond length in water. Consequently, this study provides novel insights into noble-metal-free catalyst design from the viewpoint of the internal magnetic field.

## Conflicts of interest

There are no conflicts to declare.

## Author contributions

M. M. conceived the idea and wrote the paper; Y. O. and R. F. synthesized the materials and conducted the hydrogen evolution tests and SA measurements; H. Y. reviewed and validated the work shown in the paper; and H. Y. rendered helpful discussions.

## Supplementary Material

RA-013-D2RA07865A-s001
