# Supplementary material for: Hydrogen evolution reaction following the Slater–Pauling curve: acceleration of rate processes induced from dipole interaction between protons and ferromagnetic catalysts
Source: RSC Adv. 2023 Apr 25;13(19):12941–50. doi: 10.1039/d2ra07865a (PMC10128105; doi:10.1039/d2ra07865a)
Supplement: RA-013-D2RA07865A-s001 [file RA-013-D2RA07865A-s001.pdf]

Electronic Supplementary Material (ESI) for RSC Advances.
This journal is © The Royal Society of Chemistry 2023
Please do not adjust margins

Supplementary data

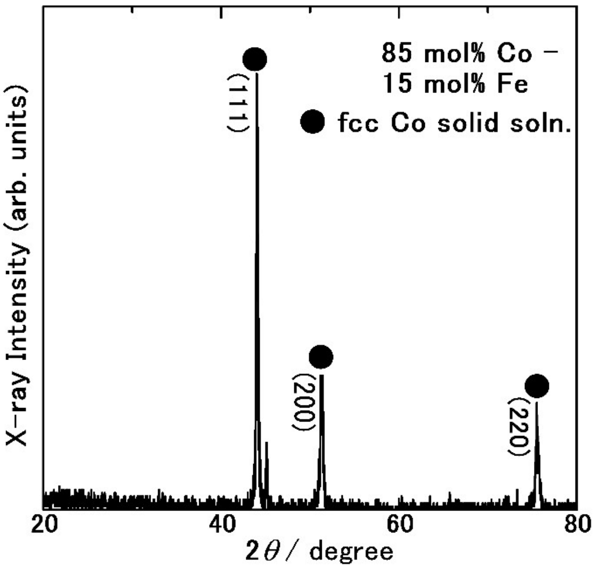

Fig.S-1 XRD pattern of 85 mol% Co – 15 mol% Fe sample composed of fcc Co solid solution.[47]

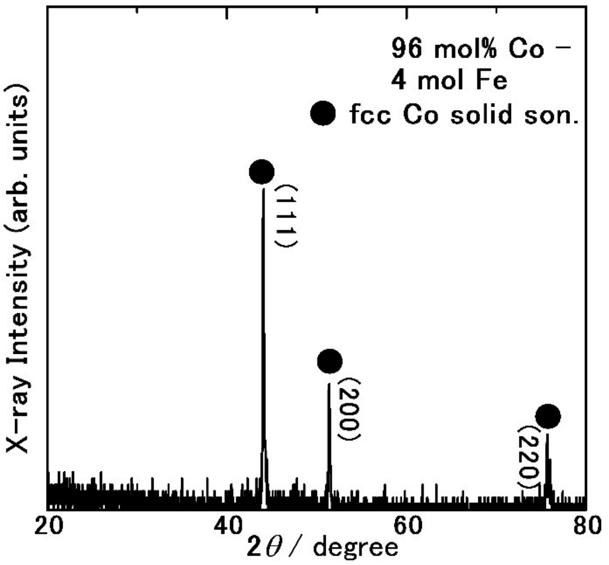

Fig.S-2 XRD pattern of 96 mol% Co – 4 mol% Fe sample composed of fcc Co solid solution. [47]

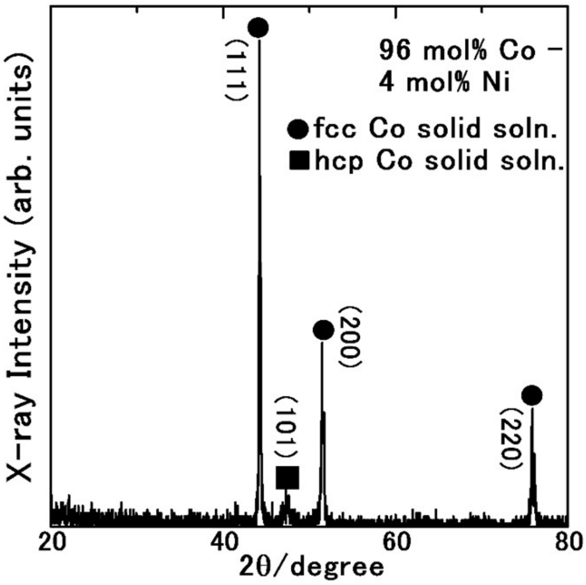

Fig.S-3 XRD pattern of 96 mol% Co – 4 mol% Fe sample composed of fcc Co solid solution.[47]

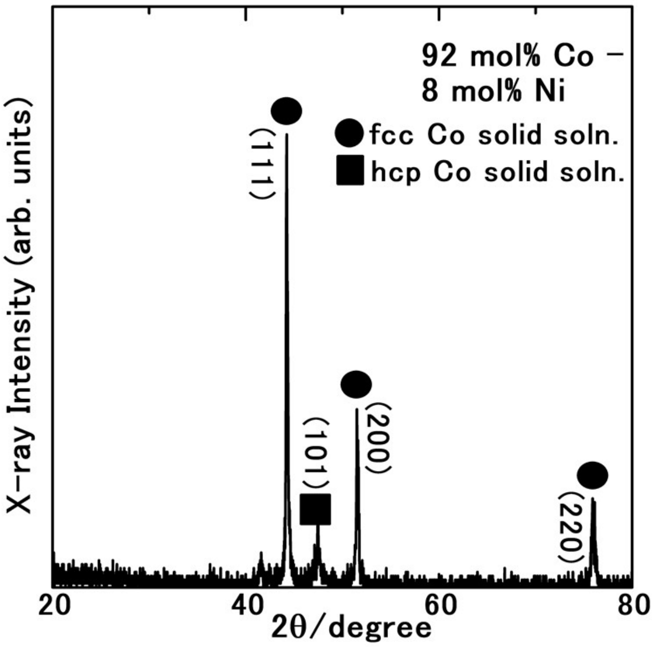

Fig.S-4 XRD pattern of 92 mol% Co – 8 mol% Ni sample composed of fcc[47] and hcp[48] Co solid solutions.

Please do not adjust margins

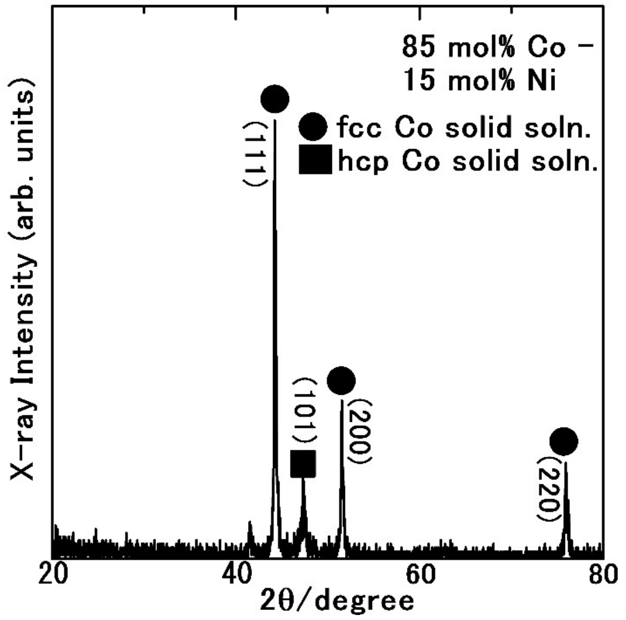

Fig.S-5 XRD pattern of 85 mol% Co – 15 mol% Ni sample composed of fcc[47] and hcp[48] Co solid solutions.

Table S1 Summary of the thermodynamic values at 298.15 K and their uncertainties, *U*, of the substances of interest.

Substances	$\Delta_f H_m^\circ$ /(kJ (mol of compd.)$^{-1}$)	$U(\Delta_f H_m^\circ)$ /(kJ (mol of compd.)$^{-1}$)	S_m° /(J K^{-1} (mol of compd.)$^{-1}$)	$U(S_m^\circ)$ /(J K^{-1} (mol of compd.)-^{1})	$\Delta_f G_m^\circ$ /(kJ (mol of compd.)$^{-1}$)	$U(\Delta_f G_m^\circ)$ /(kJ (mol of compd.)$^{-1}$)	Refs.
NH_3BH_3(cr)	-178.0	± 5.9	unknown	-	unknown	-	(37)
NH_3(aq)	-81.170	± 0.326	109.040	± 0.913	-26.673	± 0.305	(49)
NH^{4+}(aq)	-133.260	± 0.250	111.170	± 0.400	-79.398	± 0.278	(49)
$B(OH)_3$(aq)	-1072.800	± 0.800	162.400	± 0.600	-969.268	± 0.820	(49)
BO_2^-(aq)	-772.37	± 0.56*2	-37.2	± 0.25*2	-678.89	± 0.57[a]	(50)
H_2O(l)	-285.830	± 0.040	69.950	± 0.030	-237.140	± 0.041	(49)
H_2(g)	0.000	± 0.000	130.680	± 0.003	0.000	± 0.000	(49)

[a] Uncertainties of BO_2^-(aq) were estimated to be equal to those of $B(OH)_3$(aq).

This journal is © The Royal Society of Chemistry 20xx

Please do not adjust margins
